# Prognostic and Predictive Significance of Claudin-6 Expression in Advanced-Stage High-Grade Serous Ovarian Carcinoma

**DOI:** 10.3390/diagnostics16081175

**Published:** 2026-04-15

**Authors:** Teyfik Demir, Mehmet Kefeli, Ayşe Rumeysa Aydoğan Demir, Fatma Nur Uygun, Melih Akpunar, Elif Tekce Yıldız, Güzin Demirağ

**Affiliations:** 1 Department of Medical Oncology, Faculty of Medicine, Ondokuz Mayıs University, 55270 Samsun, Türkiye; dr.melihakpunar94@gmail.com (M.A.); guzindemirag@gmail.com (G.D.); 2 Department of Pathology, Faculty of Medicine, Ondokuz Mayıs University, 55270 Samsun, Türkiye; mehmetkefeli@gmail.com (M.K.); keskinfatmanur1993@gmail.com (F.N.U.); 3 Department of Obstetrics and Gynecology, Samsun City Hospital, 55090 Samsun, Türkiye; rmys.aydgn@hotmail.com; 4 Department of Internal Medicine, Faculty of Medicine, Ondokuz Mayıs University, 55270 Samsun, Türkiye; eliftekce18@gmail.com

**Keywords:** high-grade serous ovarian carcinoma, claudin-6, platinum resistance, biomarker, advanced stage

## Abstract

**Background/Objectives**: Claudin-6 (CLDN6) is an oncofetal tight junction protein that has recently emerged as a promising therapeutic target in various solid tumors. Despite this potential, the clinical significance of CLDN6 expression in advanced-stage high-grade serous ovarian carcinoma (HGSC)—specifically its role in platinum resistance—remains poorly understood. **Methods**: This retrospective study analyzed 119 patients with newly diagnosed FIGO stage III–IV HGSC who received platinum-based chemotherapy at a single tertiary center between 2015 and 2025. CLDN6 expression was evaluated via immunohistochemistry (IHC) on formalin-fixed paraffin-embedded (FFPE) tumor samples. High CLDN6 expression was defined as moderate-to-strong membranous staining in ≥50% of tumor cells. Clinicopathologic associations were assessed using chi-square tests, while logistic regression analysis identified predictors of platinum resistance. Finally, overall survival (OS) and progression-free survival (PFS) were evaluated using Kaplan–Meier methods and Cox proportional hazards models. **Results**: High CLDN6 expression was observed in 31 patients (26%). CLDN6 expression was not significantly associated with age, CA-125 level, lymph node metastasis, distant metastasis, surgical approach, or residual disease status. However, high CLDN6 expression was significantly associated with platinum resistance (61.3% vs. 28.4%, *p* = 0.001). In multivariable logistic regression analysis, residual disease (OR = 10.12, *p* > 0.001), high CLDN6 expression (OR = 4.52, *p* = 0.008), and elevated CA-125 levels (OR = 0.64, *p* = 0.041) were independently associated with platinum resistance. Median OS for the entire cohort was 43.8 months. High CLDN6 expression was associated with shorter OS (38.0 vs. 45.7 months, *p* = 0.042) and remained an independent predictor of mortality in multivariable Cox analysis (HR = 1.90, *p* = 0.026). CLDN6 expression showed a trend toward shorter PFS but did not reach statistical significance (*p* = 0.096). **Conclusions**: High CLDN6 expression is associated with platinum resistance and inferior overall survival in patients with advanced-stage HGSC. These findings suggest that CLDN6 may serve as a clinically relevant biomarker for chemoresistance and tumor aggressiveness. In the context of emerging CLDN6-targeted therapies, routine assessment of CLDN6 expression may facilitate the development of biomarker-driven therapeutic strategies for advanced ovarian cancer.

## 1. Introduction

Ovarian cancer remains one of the most lethal gynecologic malignancies worldwide and is a major cause of cancer-related mortality among women [1,2]. Despite advances in surgical management and systemic therapies, survival outcomes remain poor, particularly in advanced-stage disease. High-grade serous ovarian carcinoma (HGSC) represents the most common histologic subtype of epithelial ovarian cancer and accounts for the majority of ovarian cancer-related deaths [3,4]. Although screening strategies have been extensively evaluated, no effective population-based screening method has demonstrated a clear survival benefit [5].

The standard treatment for advanced ovarian cancer consists of cytoreductive surgery followed by platinum-based chemotherapy [6,7]. While most patients initially respond to treatment, recurrence occurs in the majority of cases, and many patients eventually develop platinum resistance. Once platinum resistance develops, therapeutic options are limited and prognosis deteriorates significantly [8,9]. The integration of immune checkpoint inhibitors and other novel systemic therapies has shown only modest clinical benefit, underscoring the need for reliable predictive and prognostic biomarkers to guide treatment strategies [10,11]. Recent studies have highlighted the importance of molecular biomarkers and gene signatures in predicting prognosis and treatment response in ovarian cancer, emphasizing the need for clinically relevant targets [12].

Tight junctions are essential components of epithelial cell polarity and barrier integrity. They are composed of transmembrane proteins, including claudins, occludins, and junctional adhesion molecules [13,14,15]. The claudin family consists of at least 27 members, several of which have been implicated in tumor progression, invasion, and metastasis. Claudin-6 (CLDN6), an oncofetal tight junction protein, has recently emerged as a molecule of considerable interest in oncology [16,17]. CLDN6 is physiologically expressed during embryonic development but is largely absent in normal adult tissues. However, aberrant re-expression has been identified in various solid tumors, including ovarian cancer [18,19]. Previous studies have suggested that CLDN6 expression in ovarian cancer is associated with tumor progression and unfavorable clinical outcomes, and it has been proposed as a potential therapeutic target, particularly in the context of emerging targeted and immunotherapeutic strategies [20,21].

Beyond its structural role in tight junctions, CLDN6 has been shown to participate in tumor-related signaling pathways regulating proliferation, apoptosis, migration, and cellular plasticity [22,23]. Antibodies targeting CLDN6 have already been engineered as bispecific single-chain antibodies, as well as anti-CLDN6 antibody–drug conjugates, for use in targeted immunotherapy [24,25]. The antibody–drug conjugate CLDN6-23-ADC (anti-CLDN6) has demonstrated a significant reduction in tumor volume in an ovarian cancer PDX model and represents a promising targeted therapeutic strategy. It is currently being evaluated in a phase I clinical trial for the treatment of CLDN6-overexpressing cancers [26].

As therapeutic strategies targeting CLDN6 continue to evolve, understanding its clinical relevance in specific ovarian cancer subtypes becomes increasingly important. However, the clinical significance of CLDN6 expression in advanced-stage HGSC, particularly in relation to platinum resistance, remains unclear. Therefore, we investigated CLDN6 expression in a relatively homogeneous and uniformly treated cohort of advanced-stage HGSC patients receiving platinum-based chemotherapy, aiming to clarify its association with platinum resistance and its prognostic implications. Such insights may help refine biological stratification within HGSC and inform future biomarker-oriented therapeutic approaches.

## 2. Materials and Methods

### 2.1. Study Population

Patients with newly diagnosed advanced-stage (FIGO stage III–IV) high-grade serous carcinoma of the ovarian, fallopian tube, or primary peritoneal origin who were followed at the Department of Medical Oncology, Ondokuz Mayıs University Faculty of Medicine, between January 2015 and December 2025 were retrospectively evaluated.

Histological classification was established according to the World Health Organization Classification of Female Genital Tumours [27]. Disease stage was assigned according to the International Federation of Gynecology and Obstetrics (FIGO) staging system [28]. In patients who underwent primary cytoreductive surgery, staging was based on surgical and pathological findings. For patients who did not undergo upfront surgery, advanced-stage disease was defined based on radiological findings consistent with advanced-stage disease (FIGO stage III–IV) in combination with histopathological confirmation obtained from biopsy specimens.

All patients received standard platinum-based chemotherapy following primary or interval debulking surgery, or as systemic treatment in patients deemed unsuitable for surgery.

The study was approved by the Institutional Ethics Committee of Ondokuz Mayıs University Faculty of Medicine (Approval No: 2025/33, Date: 12 February 2025) and was conducted in accordance with the ethical principles of the Declaration of Helsinki. The requirement for written informed consent was waived due to the retrospective nature of the study.

**Figure 1:**
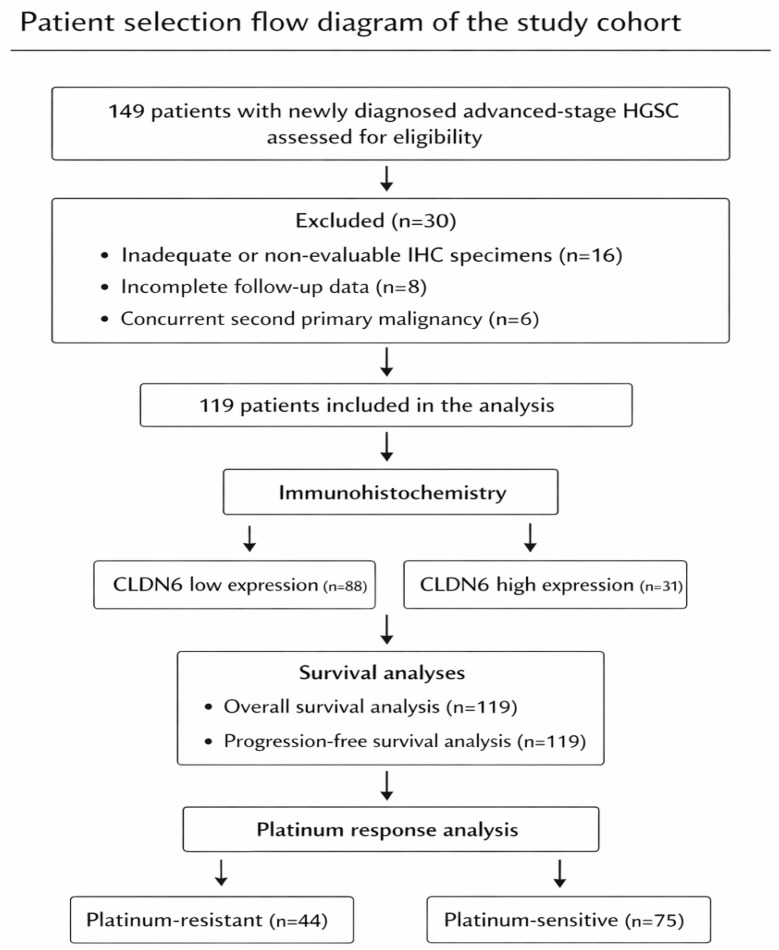
A total of 149 patients with advanced-stage HGSC were assessed, and 119 patients were included in the final analysis after exclusions. CLDN6 expression was categorized as low (*n* = 88) or high (*n* = 31), and survival and platinum response analyses were performed.

Patients with histologically confirmed advanced-stage HGSC and available tumor tissue for immunohistochemical analysis were included. A total of 149 consecutive patients were initially identified. Sixteen patients were excluded due to insufficient or unavailable tumor tissue, eight due to incomplete follow-up data, and six due to the presence of a concurrent second primary malignancy. After these exclusions, 119 patients constituted the final study cohort. The patient selection process is illustrated in Figure 1. Cases with missing key clinicopathological or survival data were excluded, and no imputation methods were applied. All analyses were conducted on complete-case data.

### 2.2. Data Collection and Clinical Variables

Demographic and clinicopathological data were retrieved from electronic medical records, including age at diagnosis, serum CA-125 level at diagnosis (IU/mL), lymph node metastasis status, presence of distant metastasis, platinum-free interval (PFI), receipt of neoadjuvant chemotherapy, and surgical approach (primary debulking surgery, interval debulking surgery, or no surgery).

Key clinical dates including the date of diagnosis, date of disease progression, date of death, and date of last follow-up. These data were used to calculate progression-free survival (PFS) and overall survival (OS). Tumor response and disease progression were assessed radiologically according to the Response Evaluation Criteria in Solid Tumors (RECIST) version 1.1 [29]. Patients were followed at approximately 3-month intervals with clinical evaluation, serum CA-125 measurement, and imaging when clinically indicated.

OS was defined as the time from the date of diagnosis to death from any cause. Patients alive at the time of analysis were censored at the last follow-up date. PFS was defined as the time from diagnosis to radiologic or clinical progression or death, whichever occurred first. Patients without events were censored at the last follow-up.

Platinum resistance was defined as disease recurrence within 6 months after completion of platinum-based chemotherapy, whereas recurrence ≥ 6 months was classified as platinum-sensitive disease. PFI was defined as the interval from the last platinum-based chemotherapy cycle to documented disease recurrence. Platinum resistance analysis was restricted to patients who experienced recurrence after first-line platinum-based chemotherapy therapy.

### 2.3. Treatment Strategy

All patients were evaluated by a multidisciplinary tumor board including a gynecologic oncologist to determine surgical operability. Patients suitable for primary cytoreduction underwent primary debulking surgery followed by platinum-based chemotherapy.

Patients unlikely to achieve optimal cytoreduction due to extensive disease distribution on imaging or poor performance status received neoadjuvant platinum-based chemotherapy. After completion of neoadjuvant therapy, patients were re-evaluated for interval debulking surgery. Those demonstrating adequate clinical and radiologic response underwent interval cytoreduction, whereas patients who remained inoperable continued systemic therapy.

Residual disease status was determined based on operative and postoperative surgical reports. Complete cytoreduction (R0) was defined as the absence of any macroscopic residual tumor at the end of surgery. Incomplete cytoreduction (R+) was defined as the presence of any visible residual disease. Residual disease assessment was performed at the completion of either primary or interval debulking surgery.

### 2.4. Immunohistochemistry and Evaluation of Claudin-6 Expression

Immunohistochemical analysis was performed on formalin-fixed paraffin-embedded tumor tissues obtained from either surgical specimens or diagnostic biopsy samples of the primary tumor. Archived tumor specimens from patients diagnosed with HGSC were re-evaluated using hematoxylin and eosin (H&E) staining to confirm the diagnosis and select representative tumor areas. Formalin-fixed, paraffin-embedded (FFPE) tissue blocks were retrieved, and 4-μm thick sections were cut and mounted on adhesive-coated slides.

Immunohistochemical staining was performed using a fully automated immunostaining platform (Ventana Benchmark Ultra; Roche Diagnostics, Basel, Switzerland). Sections were incubated with a mouse monoclonal anti-Claudin-6 antibody (Clone E7U2O, 1:150 dilution; Cell Signaling Technology, Danvers, MA, USA) according to the manufacturer’s protocol. Appropriate positive and negative controls were included in each staining run.

Only membranous staining was considered indicative of CLDN6 expression. Immunostained slides were independently evaluated by one experienced gynecologic pathologist who was blinded to clinical data and survival outcomes.

Staining intensity was graded as 0 (absent), 1+ (weak), 2+ (moderate), or 3+ (strong), and the percentage of positive tumor cells was recorded.

**Figure 2:**
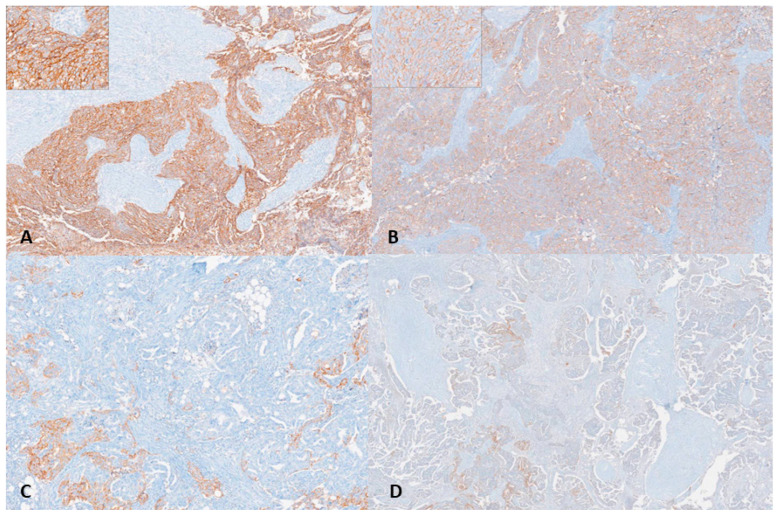
Claudin-6 immunohistochemical expression in tumor samples. High Claudin-6 expression, demonstrating diffuse, moderate-to-strong (2+–3+) membranous staining in ≥50% of tumor cells (**A**,**B**; insets show higher magnification). Low Claudin-6 expression, showing absent to weak (0–1+) staining and/or expression in <50% of tumor cells (**C**,**D**).

Claudin-6 expression was dichotomized using a predefined combined intensity–percentage scoring approach. Tumors demonstrating moderate-to-strong membranous staining (2+ or 3+) in ≥50% of tumor cells were classified as high expression. All other cases (0–1+ intensity and/or <50% positive tumor cells) were categorized as low expression [30]. Representative images of Claudin-6 immunohistochemical staining are shown in Figure 2.

### 2.5. Statistical Analysis

The optimal cut-off value for baseline CA-125 in predicting mortality was determined using receiver operating characteristic (ROC) curve analysis. The cut-off value was selected based on the Youden index. Baseline CA-125 levels were dichotomized according to the ROC-derived cut-off value (714.5 IU/mL). Age was dichotomized using the cohort median value (62 years) and categorized as ≤62 and >62 years for subsequent analyses.

The primary endpoint was the association between CLDN6 expression and platinum resistance. Secondary endpoints included OS and PFS.

All statistical analyses were performed using IBM SPSS Statistics software (version 25.0; IBM Corp., Armonk, NY, USA). All tests were two-sided, and a *p* < 0.05 was considered statistically significant.

Continuous variables were assessed for normality using the Kolmogorov–Smirnov test and were expressed as mean ± standard deviation (SD) or median (interquartile range [IQR]), as appropriate. Categorical variables were summarized as frequencies and percentages.

Associations between CLDN6 expression and clinicopathological characteristics were evaluated using the Pearson chi-square test or Fisher’s exact test.

Follow-up duration was defined as the time from diagnosis to death or last follow-up. The median follow-up time was estimated using the reverse Kaplan–Meier method.

Survival analyses were performed for OS and PFS, as defined above. Survival curves were estimated using the Kaplan–Meier method and compared using the log-rank test. Median survival times with 95% confidence intervals (CIs) were reported.

The prognostic significance of CLDN6 expression and other clinicopathological variables was evaluated using Cox proportional hazards regression models. Multivariate Cox regression models were constructed by including variables considered clinically relevant together with those showing *p* < 0.10 in univariate analysis. Hazard ratios (HRs) and 95% CIs were calculated. The proportional hazards assumption was assessed by inspection of log-minus-log survival plots. PFI was not included in the OS analysis due to its inherent correlation with disease progression and its potential to introduce collinearity. The proportional hazards assumption was evaluated using Schoenfeld residuals and the cox.zph function in R.

To further examine the association between CLDN6 expression and platinum resistance, binary logistic regression analysis was performed, and odds ratios (ORs) with 95% CIs were reported. No missing data were identified for the variables included in the analyses.

## 3. Results

A total of 119 patients with newly diagnosed advanced-stage high-grade serous ovarian carcinoma were included in the analysis. The median follow-up duration was 63 months (95% CI: 57.9–68.1). Based on immunohistochemical evaluation, high CLDN6 expression was observed in 31 patients (26%), while the majority of tumors (74%) showed low CLDN6 expression. The clinicopathological characteristics of the study cohort according to CLDN6 expression are summarized in Table 1. CLDN6 expression was not significantly associated with age group (*p* = 0.643), CA-125 level (*p* = 0.159), lymph node metastasis (*p* = 0.991), distant metastasis (*p* = 0.492), receipt of neoadjuvant chemotherapy (*p* = 0.763), surgical approach (*p* = 0.311), or residual disease after surgery (*p* = 0.856). However, high CLDN6 expression was significantly associated with platinum resistance (61.3% vs. 28.4%, *p* = 0.001).

As shown in Table 2, residual disease after surgery and high CLDN6 expression were independently associated with platinum resistance. Patients with high CLDN6 expression had a significantly increased risk of platinum resistance (OR = 4.52, 95% CI: 1.49–13.68, *p* = 0.008). Similarly, the presence of residual disease was strongly associated with platinum resistance (OR = 10.12, 95% CI: 2.87–35.74, *p* < 0.001). CA-125 levels were also significantly associated with platinum resistance (OR = 0.64, 95% CI: 0.42–0.98, *p* = 0.041). In contrast, lymph node metastasis, distant metastasis, and age were not independently associated with platinum resistance.

**Figure 3:**
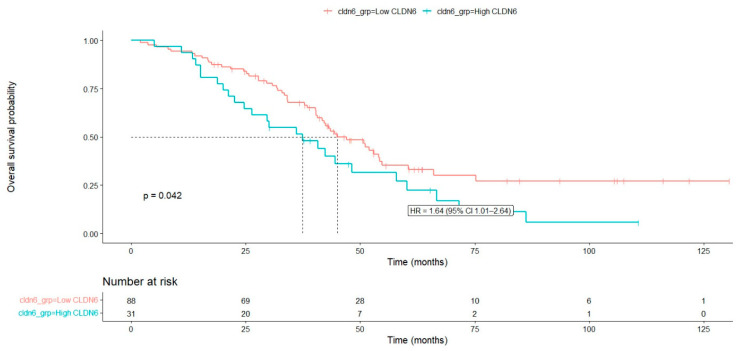
Kaplan–Meier overall survival (OS) curves according to Claudin-6 expression. Patients with high Claudin-6 expression exhibited shorter overall survival compared to those with low expression; however, the difference was not statistically significant (log-rank *p* = 0.42).

Overall survival analysis according to clinicopathologic variables is summarized in Table 3. The median overall survival for the entire cohort was 43.8 months (95% CI: 38.05–49.54). High CLDN6 expression was significantly associated with poorer overall survival; patients with high CLDN6 expression had shorter survival compared with those with low expression (median OS = 38.0 vs. 45.7 months, *p* = 0.042) (Figure 3). Elevated CA-125 levels were also associated with poorer survival (*p* = 0.028). The presence of distant metastasis significantly affected survival (*p* = 0.016). Surgical approach was strongly associated with prognosis, with patients who did not undergo surgery demonstrating markedly worse survival (median OS: 20.4 months, *p* < 0.001). Residual disease after surgery was also significantly associated with poorer survival (*p* = 0.004). Age, lymph node metastasis, and receipt of neoadjuvant chemotherapy were not significantly associated with overall survival.

Multivariable Cox proportional hazards regression analysis was performed to identify independent prognostic factors for overall survival (Table 4). High CLDN6 expression was significantly associated with increased mortality risk. Patients with high CLDN6 expression had a 1.9-fold increased risk of death compared with those with low CLDN6 expression (HR = 1.90, 95% CI: 1.08–3.36, *p* = 0.026). Similarly, elevated CA-125 levels (≥714.50) were independently associated with worse overall survival (HR = 1.87, 95% CI: 1.07–3.25, *p* = 0.027). Although distant metastasis showed a trend toward increased mortality risk (HR = 1.82), this association did not reach statistical significance (*p* = 0.144). Surgical approach was not significantly associated with overall survival in the multivariable model (*p* = 0.377). In contrast, the presence of residual disease after surgery was identified as an independent adverse prognostic factor. Patients with residual tumor had a 2.24-fold higher risk of death compared with those without residual disease (HR = 2.24, 95% CI: 1.26–3.96, *p* = 0.005). Taken together, these findings indicate that CLDN6 expression is associated with platinum resistance and poorer overall survival in patients with advanced-stage HGSC. No statistically significant violation of the proportional hazards assumption was observed (global test *p* = 0.053).

PFS was analyzed using the Kaplan–Meier method, and the results are summarized in Table 5. In the overall cohort, the 2-year and 5-year PFS rates were 28.3% and 12.6%, respectively, with a median PFS of 15.73 months. CLDN6 expression showed a trend toward shorter PFS in the high-expression group; however, the difference did not reach statistical significance (*p* = 0.096). Similarly, age, CA-125 level, lymph node metastasis, and receipt of neoadjuvant chemotherapy were not significantly associated with PFS. In contrast, the presence of distant metastasis was significantly associated with poorer PFS (*p* = 0.021). Surgical approach also had a significant impact on PFS (*p* = 0.009), with patients who did not undergo surgery demonstrating the shortest PFS. Furthermore, residual disease after surgery was strongly associated with worse PFS (*p* = 0.001). Taken together, these findings indicate that CLDN6 expression is associated with platinum resistance and poorer overall survival in patients with advanced-stage HGSC.

## 4. Discussion

In this study, we sought to clarify the clinical relevance of CLDN6 expression in advanced-stage HGSC, a setting in which reliable biomarkers for platinum resistance remain limited. Our findings demonstrate that high CLDN6 expression is significantly associated with platinum resistance in logistic regression analysis and is independently predicted to reduce OS in multivariable Cox models. Importantly, CLDN6 expression was not significantly correlated with parameters reflecting tumor burden or anatomical dissemination, suggesting that its clinical impact may reflect intrinsic tumor aggressiveness rather than disease extent. Collectively, these findings suggest that CLDN6 may represent a clinically relevant biomarker of treatment resistance and poor prognosis in advanced HGSC.

Previous studies have reported a correlation between CLDN6 overexpression and poor survival outcomes in ovarian and other gynecologic malignancies [31,32]. Yuceer et al. demonstrated that elevated CLDN6 expression was independently associated with reduced OS and PFS in serous ovarian cancer [33]. Similarly, Gao et al. reported that CLDN6 overexpression predicted unfavorable overall survival and was associated with alterations in the tumor immune microenvironment [34]. Our findings are consistent with these reports in terms of OS, further supporting the link between CLDN6 expression and aggressive tumor behavior. However, prior studies generally included relatively small sample sizes and heterogeneous patient populations encompassing multiple histological subtypes and disease stages. In contrast, the present study specifically focused on a homogeneous cohort of advanced-stage HGSC, thereby minimizing biological and clinical heterogeneity. Interestingly, unlike some previous reports, we did not observe a significant association between CLDN6 expression and progression-free survival. This discrepancy may be attributable to differences in cohort composition, scoring methodologies, stage distribution, and treatment strategies across studies.

Earlier investigations, including the study by Wang et al., suggested that CLDN6 expression may be associated with increased metastatic potential [31]. Similarly, studies in cervical adenocarcinoma have reported correlations between CLDN6 overexpression and adverse pathological features such as lymphovascular space invasion and nodal metastasis [35]. In contrast, our study did not demonstrate a significant association between CLDN6 expression and lymph node involvement, distant metastasis, or residual disease status. These discrepancies may reflect differences in stage distribution, sample size, histological heterogeneity, or variability in immunohistochemical scoring and cut-off definitions across studies. Importantly, our cohort was restricted to advanced-stage HGSC, a setting in which metastatic dissemination is already prevalent, potentially attenuating detectable differences in anatomical spread according to CLDN6 expression status. This finding suggests that CLDN6 expression may reflect intrinsic tumor aggressiveness rather than the extent of anatomical dissemination.

A key finding of our study is the strong and independent association between high CLDN6 expression and platinum resistance, which remained significant after multivariable adjustment. Given that platinum resistance is a major determinant of survival in advanced ovarian cancer, identifying biomarkers capable of stratifying chemoresistant disease is of substantial clinical importance. While previous studies have primarily emphasized the prognostic implications of CLDN6, limited data have explored its relationship with treatment response [36]. Mechanistic insights from other gynecologic malignancies provide a biologically plausible explanation for this association. In cervical adenocarcinoma, CLDN6 overexpression has been shown to enhance tight junction barrier function by suppressing paracellular flux of small molecules, potentially limiting intratumoral drug penetration. Moreover, CLDN6 upregulation was associated with increased expression of aldo–keto reductase family proteins, which are known to metabolize chemotherapeutic agents and have been implicated in cisplatin resistance [35]. These findings suggest that CLDN6 may promote chemotherapy resistance through dual mechanisms, including reduced drug penetration across tumor cell junctions and activation of drug-detoxifying pathways. Although these mechanisms were not directly investigated in our cohort of advanced-stage HGSC, they provide a plausible biological explanation for the observed association between CLDN6 overexpression and platinum resistance. Notably, despite its association with platinum resistance and overall survival, CLDN6 expression was not significantly correlated with lymph node involvement, distant metastasis, or residual disease status. This pattern suggests that CLDN6 may not primarily influence anatomical dissemination but rather contribute to tumor aggressiveness through molecular and functional pathways related to chemoresistance. Taken together, these observations support the concept that CLDN6 represents a marker of intrinsic biological aggressiveness rather than tumor burden in advanced-stage HGSC.

Given its restricted expression in normal adult tissues and preferential overexpression in malignant cells, CLDN6 has emerged as an attractive therapeutic target [21]. CLDN6-directed antibody–drug conjugates (ADCs), bispecific antibodies, and CAR-T cell therapies have demonstrated encouraging preclinical and early-phase clinical activity in solid tumors [37,38]. In this context, our observation linking CLDN6 overexpression to platinum resistance provides additional clinical relevance to ongoing efforts targeting this molecule. In advanced-stage HGSC, where therapeutic options for platinum-resistant disease remain limited, CLDN6-targeted strategies may represent a promising avenue for treatment development. Furthermore, CLDN6 expression may serve as a potential stratification biomarker to identify patients who are less likely to benefit from conventional platinum-based therapy and who may be candidates for novel targeted or immune-based therapeutic approaches. Prospective validation in biomarker-driven clinical trials will be essential to define the therapeutic utility of CLDN6-directed strategies in ovarian cancer.

One of the major strengths of the present study is the inclusion of a relatively homogeneous cohort of advanced-stage HGSC patients treated with uniform platinum-based chemotherapy, which reduces clinical heterogeneity and allows a more reliable assessment of the biological impact of CLDN6 expression. Several limitations of this study should be acknowledged. First, its retrospective and single-center design may introduce inherent selection bias and limit the generalizability of the findings, particularly as patients with missing clinical or follow-up data were excluded from the analysis. Second, although our cohort was restricted to a biologically homogeneous population of advanced-stage HGSC, the sample size remains moderate and warrants validation in larger, multicenter studies. Third, the mechanistic basis underlying the association between CLDN6 expression and platinum resistance was not directly investigated. While experimental data from other tumor models provide plausible explanations, functional studies specifically in HGSC are needed to clarify the molecular pathways involved. Additionally, the impact of maintenance therapies, including bevacizumab and PARP inhibitors, was not specifically analyzed and may have influenced survival outcomes. Finally, although predefined literature-based cut-off values were applied, immunohistochemical assessment of CLDN6 lacks standardized scoring criteria, which may contribute to inter-study variability. Prospective validation studies incorporating biomarker-driven therapeutic stratification will be essential to confirm the clinical utility of CLDN6 in advanced ovarian cancer.

## 5. Conclusions

High CLDN6 expression was significantly associated with platinum resistance and poorer overall survival in patients with advanced-stage HGSC. These findings suggest that CLDN6 may represent a biomarker reflecting intrinsic tumor aggressiveness and an increased probability of treatment resistance rather than anatomical disease extent. These findings add to the growing body of evidence supporting the prognostic and predictive value of CLDN6 in ovarian cancer and highlight its potential role in biomarker-driven patient stratification. Given the ongoing development of CLDN6-targeted therapies, assessment of CLDN6 expression may have meaningful clinical implications. Further prospective and mechanistic studies are warranted to validate these findings.

## Figures and Tables

**Table 1:** Clinicopathologic characteristics according to CLDN6 expression.

Variable	CLDN6 Low (*n* = 88)	CLDN6 High (*n* = 31)	*p*-Value
**Age**			0.643
≤62 years	44 (50.0%)	17 (54.8%)	
>62 years	44 (50.0%)	14 (45.2%)	
**CA-125 level**			0.159
<714.5 U/mL	41 (46.6%)	19 (61.3%)	
≥714.5 U/mL	47 (53.4%)	12 (38.7%)	
**Lymph node metastasis**			0.991
Absent	51 (58.0%)	18 (58.1%)	
Present	37 (42.0%)	13 (41.9%)	
**Distant metastasis**			0.492
Absent	78 (88.6%)	26 (83.9%)	
Present	10 (11.4%)	5 (16.1%)	
**Neoadjuvant chemotherapy**			0.763
No	37 (42.0%)	14 (45.2%)	
Yes	51 (58.0%)	17 (54.8%)	
**Surgical approach**			0.311
No surgery	9 (10.2%)	6 (19.4%)	
Primary debulking surgery	37 (42.0%)	14 (45.2%)	
Interval debulking surgery	42 (47.8%)	11 (35.4%)	
Residual disease *			0.856
No residual tumor	30 (38.0%)	10 (40.0%)	
Residual tumor present	49 (62.0%)	15 (60.0%)	
**PFI**			**0.001**
Platinum-resistant	25 (28.4%)	19 (61.3%)	
Platinum-sensitive	63 (71.6%)	12 (38.7%)	

* Residual disease was evaluated among surgically treated patients (*n* = 104). Values are presented as *n* (%). *p* values were calculated using the chi-square test or Fisher’s exact test, as appropriate. CLDN6, Claudin-6; CA-125, cancer antigen 125; PFI, platinum-free interval. Bold values indicate statistically significant results (*p* < 0.05).

**Table 2:** Multivariable logistic regression analysis of factors associated with platinum resistance.

Variable	Odds Ratio (OR)	95% CI	*p*-Value
High CLDN6 expression	4.52	1.49–13.68	0.008
Lymph node metastasis	0.81	0.31–2.12	0.672
Distant metastasis	3.58	0.79–16.14	0.097
Residual disease	10.12	2.87–35.74	<0.001
CA-125 level	0.64	0.42–0.98	0.041
Age	1.04	0.99–1.08	0.135

OR, odds ratio; CI, confidence interval; CLDN6, Claudin-6; CA-125, cancer antigen 125.

**Table 3:** Overall survival according to clinicopathologic variables.

Variable	Median OS (Months)	95% CI	*p*-Value
Overall	43.80	38.05–49.54	**—**
**Age**			0.326
<62 years	44.06	39.78–48.35	
≥62 years	43.06	29.74–56.38	
**CLDN6 expression**			**0.042**
Low	45.73	36.75–54.71	
High	38.00	23.90–52.09	
**CA-125 level**			**0.028**
<714.5 U/mL	51.83	35.31–68.35	
≥714.5 U/mL	42.26	37.11–47.42	
**Lymph node metastasis**			0.999
Absent	45.73	38.12–53.34	
Present	42.73	39.01–46.45	
**Distant metastasis**			**0.016**
Absent	45.73	37.80–53.66	
Present	26.03	4.38–47.67	
Neoadjuvant chemotherapy			0.318
No	45.16	31.98–58.35	
Yes	42.96	37.36–48.56	
**Surgical approach**			**<0.001**
No surgery	20.36	6.84–33.88	
Primary debulking surgery	45.16	31.98–58.35	
Interval debulking surgery	48.93	40.37–57.39	
**Residual disease**			**0.004**
No residual tumor	67.63	43.92–91.34	
Residual tumor present	42.73	36.36–49.10	

*p* values were calculated using the log-rank test. CI, confidence interval; OS, overall survival; CLDN6, Claudin-6; CA-125, cancer antigen 125. Bold values indicate statistically significant results (*p* < 0.05).

**Table 4:** Multivariable Cox regression analysis for overall survival.

Variable	Hazard Ratio (HR)	95% CI	*p*-Value
**CLDN6 expression**			**0.026**
CLDN6 low	Reference		
CLDN6 high	1.90	1.08–3.36	
**CA-125 level**			**0.027**
<714.50	Reference		
≥714.50	1.87	1.07–3.25	
**Distant metastasis**			0.144
Absent	Reference		
Present	1.82	0.8–4.08	
**Surgical approach**			0.377
No surgery after neoadjuvant therapy	Reference		
Primary cytoreductive surgery	1.16	0.98–2.35	0.300
Interval debulking surgery	1.93	0.37–2.41	0.365
**Residual disease**			**0.005**
No residual tumor	Reference		
Residual tumor present	2.24	1.26–3.96	

HR, hazard ratio; CI, confidence interval; CLDN6, Claudin-6; CA-125, cancer antigen 125. *p*-values were derived from the multivariable Cox proportional hazards model. Bold values indicate statistically significant results (*p* < 0.05).

**Table 5:** Progression-free survival according to clinicopathologic variables.

Variable	2-Year PFS (%)	5-Year PFS (%)	Median PFS (Months) (95% CI)	*p*-Value
**Overall**	28.3	12.6	15.73 (14.63–16.82)	
**Age**				0.724
<62	25.7	13.0	15.33 (13.86–17.28)	
≥62	32.1	12.5	16.13 (12.93–19.33)	
**CLDN6 expression**				0.096
CLDN6 low	31.6	16.6	15.90 (14.15–17.64)	
CLDN6 high	19.4	3.2	15.60 (11.94–19.25)	
**CA-125 level**				0.093
<714.50	28.8	20.7	16.16 (14.06–18.26)	
≥714.50	27.9	5.2	15.33 (12.97–17.69)	
**Lymph node metastasis**				0.770
Absent	29.0	13.7	15.90 (14.73–17.06)	
Present	27.4	11.6	15.60 (14.09–17.10)	
**Distant metastasis**				**0.021**
Absent	30.5	13.4	16.13 (14.66–17.60)	
Present	13.3	6.7	10.73 (8.71–12.75)	
**Neoadjuvant chemotherapy**				0.243
No	32.8	17.3	15.90 (11.96–19.84)	
Yes	25.0	9.6	15.73 (14.65–16.81)	
**Surgical approach**				**0.009**
No surgery after neoadjuvant therapy	13.3	–	9.06 (5.82–12.30)	
Primary cytoreductive surgery	32.8	17.3	15.90 (11.96–19.84)	
Interval debulking surgery	28.3	12.3	16.06 (14.71–17.42)	
**Residual disease**				**0.001**
No residual tumor	45.0	26.7	19.36 (15.02–23.70)	
Residual tumor present	21.4	7.2	14.00 (12.10–15.89)	

PFS, progression-free survival; CI, confidence interval; CLDN6, Claudin-6; CA-125, cancer antigen 125. *p*-values were calculated using the log-rank test. Bold values indicate statistically significant results (*p* < 0.05).

## Data Availability

The data presented in this study are available from the corresponding author upon reasonable request. The data are not publicly available due to privacy and ethical restrictions.
